# Supplementary material for: Functional annotation of genetic associations by transcriptome-wide association analysis provides insights into neutrophil development regulation
Source: Commun Biol. 2020 Dec 18;3:790. doi: 10.1038/s42003-020-01527-7 (PMC7749173; doi:10.1038/s42003-020-01527-7)
Supplement: Supplementary file 1 — Supplementary Information [file 42003_2020_1527_MOESM1_ESM.pdf]

Supplementary materials for

“Functional annotation of genetic associations by transcriptome-wide association analysis provides insights into neutrophil development regulation”

Yao Yao, Jia Yang, Qian Qin, Chao Tang, Zhidan Li, Li Chen, Kailong Li, Chunyan Ren, Lu Chen, Shuquan Rao

This file includes:
Supplementary Figure 1-9
References

Other Supplementary Materials for this manuscript include the following:
Supplementary Data 1-5.

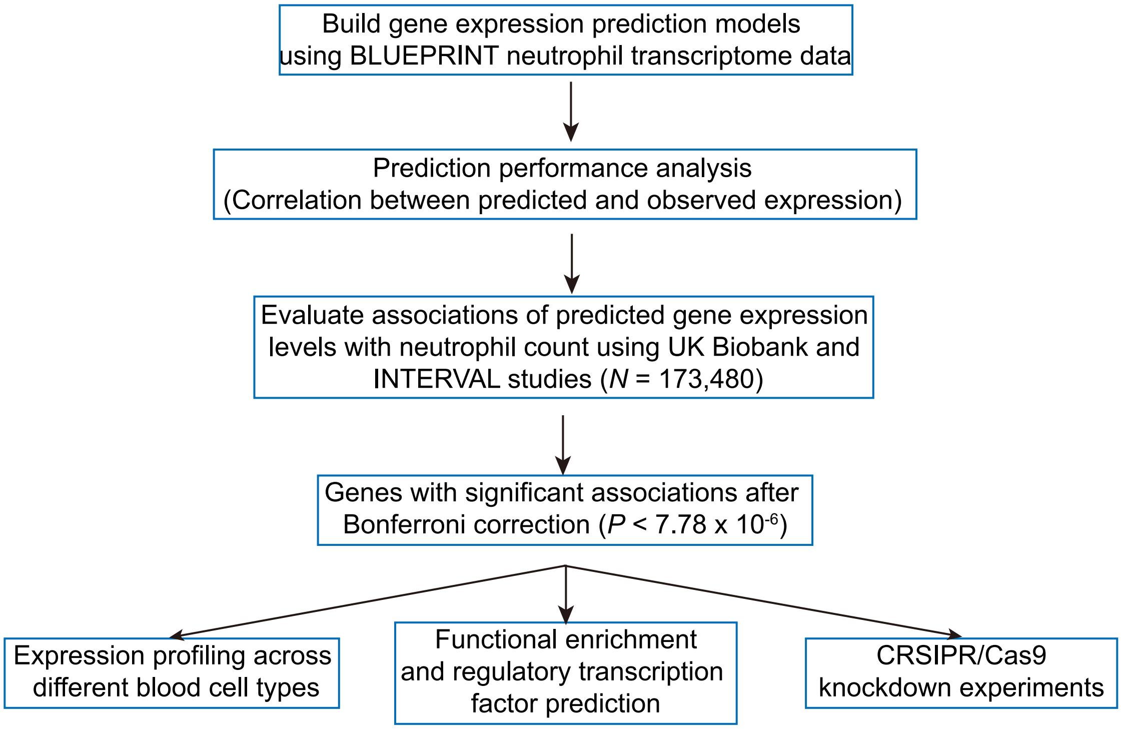

Supplementary Fig. 1 Study design flow chart

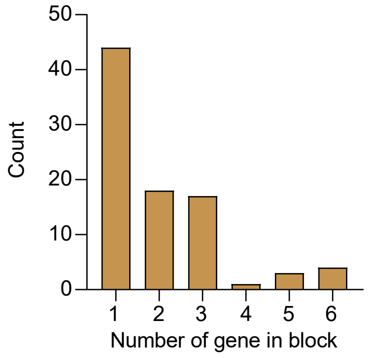

Supplementary Fig. 2 Number of Fusion TWAS hit genes (NEUT#) per locus after 1 Mb clumping

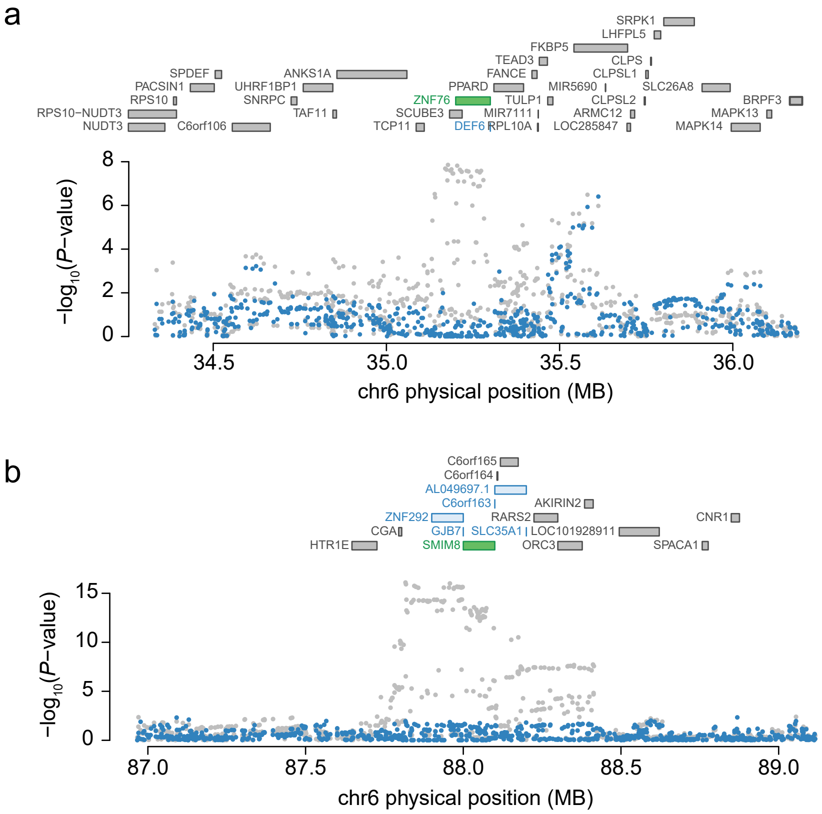

Supplementary Fig. 3 Regional association of TWAS hits with NEUT#

(a) and (b) showed two different regions with multiple TWAS hits. The top panel in each plot highlights all genes in the region. The marginally associated TWAS genes are shown in blue and the jointly significant genes are shown in green. The bottom panel shows a regional Manhattan plot of the GWAS associations for each SNP before (gray) and after (blue) conditioning on the predicted expression of the green genes. Two-sided *P*-value computed from GWAS statistics.

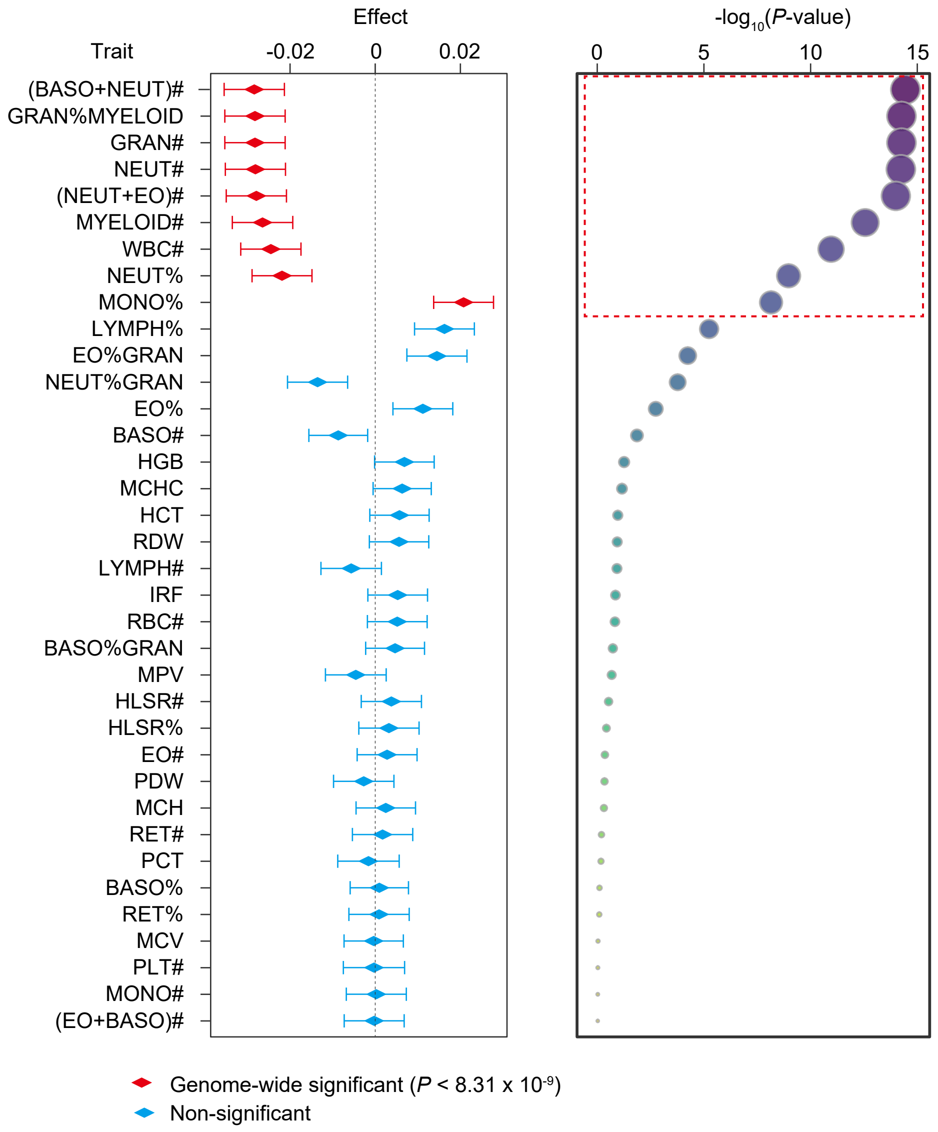

Supplementary Fig. 4 Associations of lead SNP rs11745591 at chromosome 5q13.2 with 36 blood cell indices

(a) A forest plot showing the effect size from associations of rs11745591 with 36 blood cell indices. Red colored diamonds represent the genome-wide significant SNP-trait association ($P < 8.31E\text{-}09$), while blue colored diamonds denoted non-significant results. Each diamond/circle represents the estimated effect size with 95% confidence interval.

(b) *P*-value of SNP-trait associations. For best representation of the results, $-\log_{10}(P\text{-values})$ are reported on a linear scale for best representation of the results. Dots size is in linear correlation with $-\log_{10}(P\text{-values})$. All data are from Astle *et al.* study[1].

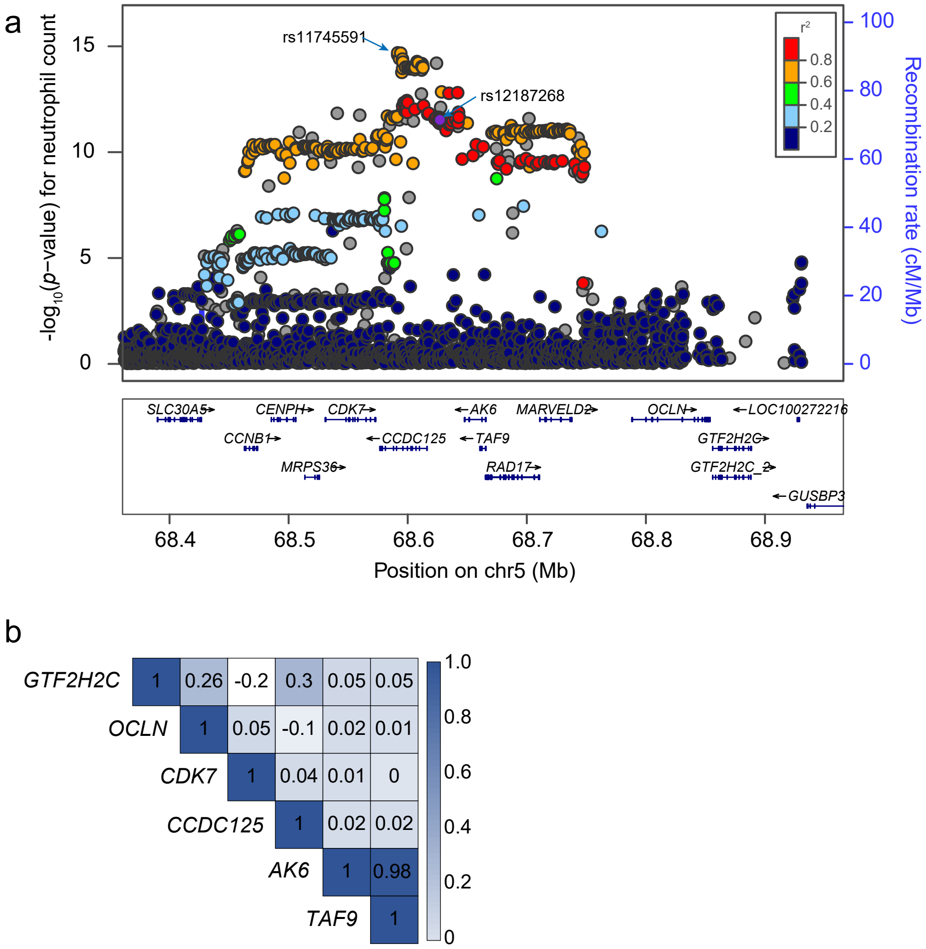

Supplementary Fig. 5 Strong LD among SNPs and target gene expression correlation at chromosome 5q13.2

(a) Regional Manhattan Plots for the NEUT# GWAS within 300 kb surrounding the *TAF9* gene at chromosome 5q13.2

Genotyped and imputed SNPs passing quality control measures are plotted with their *P* values (as $-\log_{10}$(*P*-values)) according to their chromosomal positions. Each circle represents one SNP. GWAS-lead SNP rs11745591 and TWAS-lead eQTL rs12187268 for *TAF9* and *AK6* were indicated by arrow. The color of each circle indicated the range of pairwise r^2 value with rs12187268. The gene positions and transcriptional directions are annotated in the lower panel. All data are from Astle *et al.* study[1].

(b) Total gene expression correlation (*corr.*) matrix among TWAS hits at chromosome 5q13.2.

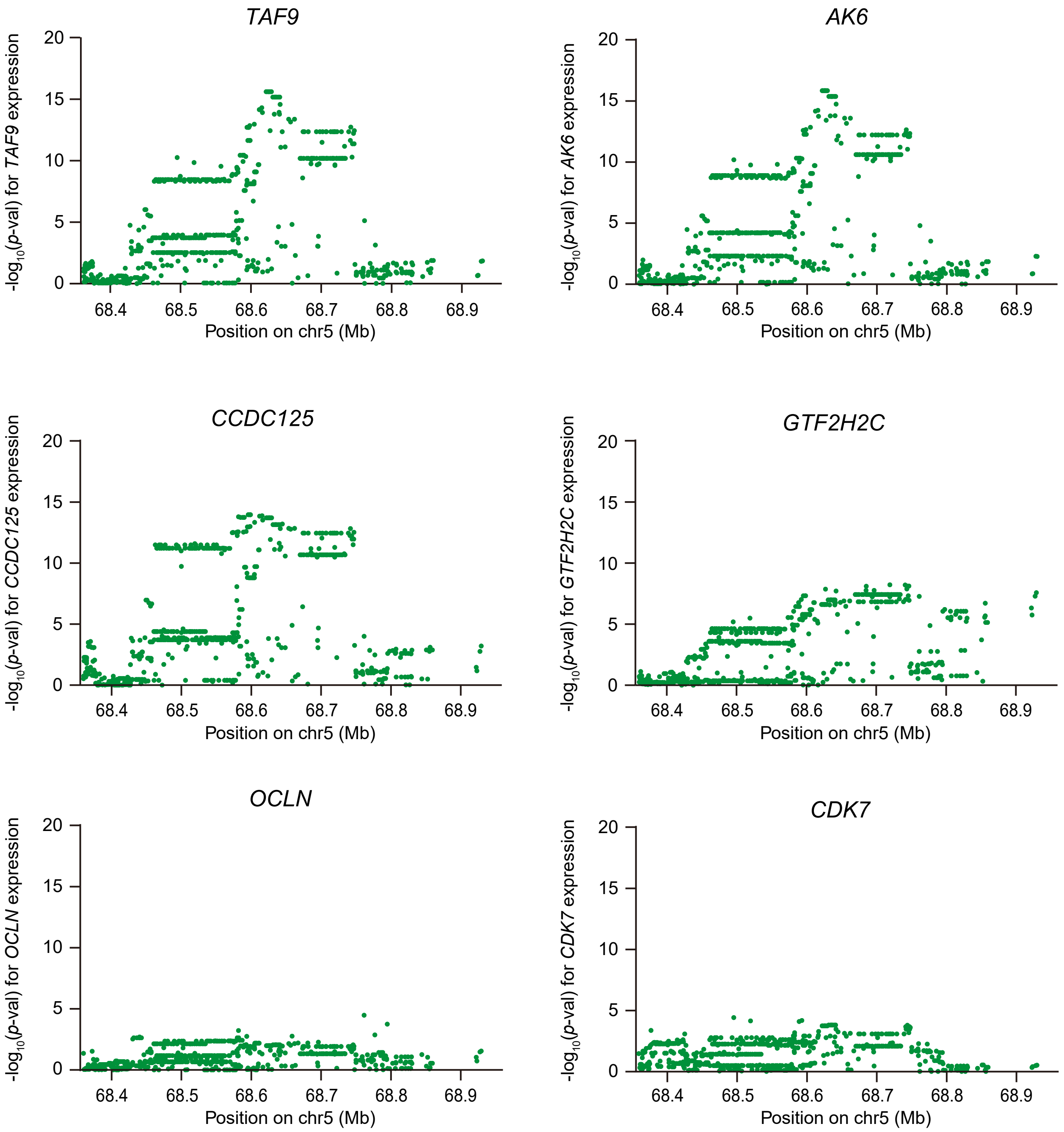

Supplementary Fig. 6 Regional cis-eQTL analysis of SNPs on TWAS hits (NEUT#) at chromosome 5q13.2

Plots show position of SNPs on the x-axis (chromosome length, Mb) and -$\log_{10}$(P-value) for SNP-gene expression associations on the y-axis shows. Genotyping and gene expression data from $CD16^{+}$ neutrophils (N = 196) were from our previous study[2]. eQTLs were identified by using linear regression model and additive genotype effects.

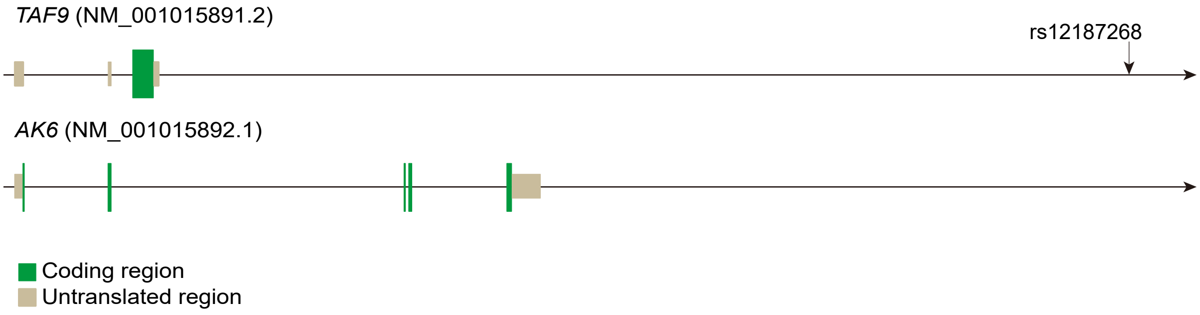

Supplementary Fig. 7 Schematic of genomic positions of *TAF9* and *AK6*

TAF9 and *AK6* shared the same 5’ regulatory region but distinct coding sequence (green).

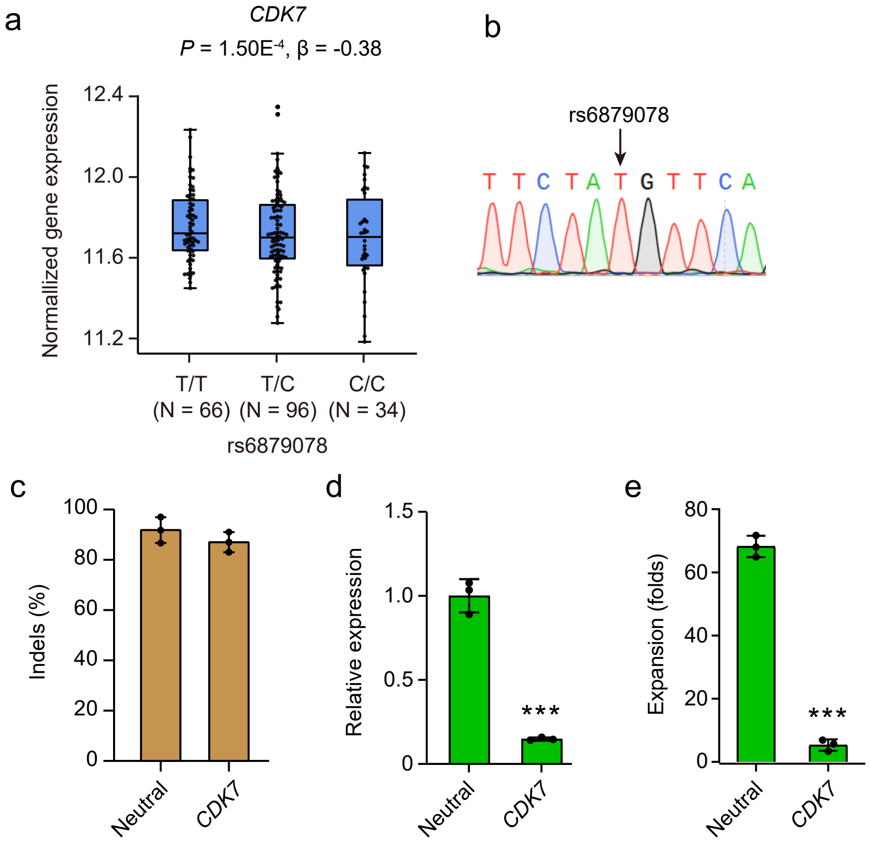

Supplementary Fig. 8 *CDK7* is indispensable for human cell viability

(a) Expression quantitative loci (eQTL) analysis of rs6879078 on *CDK7* expression. Genotyping and gene expression data from $CD16^+$ neutrophils (N = 196) were from our previous study [2].

(b) Genomic DNA Sanger sequencing traces show the genotypes of eQTLs (indicated by arrow).

(c) Editing efficiency in HSPCs following 3xNLS-SpCas9:sgRNA electroporation with indicated sgRNA. Gene edits were measured 6 days after electroporation by Sanger sequencing analysis. $N = 3$ biological replicates for each group.

(d) Expression level (mRNA) of *CDK7* from RNP edited $CD34^+$ HSPCs 4 days after neutrophil differentiation. mRNA level was determined by RT–qPCR (n = 3 biological replicates).

*** $P < 0.001$.

(e) Expansion of total cells 12 days after electroporation (n = 3 biological replicates).

*** $P < 0.001$.

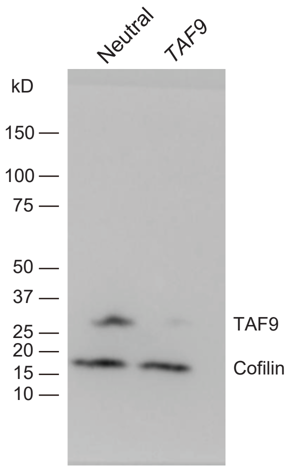

Supplementary Fig. 9 Uncropped blot images.

Supplementary references

1 Astle, W. J. *et al.* The Allelic Landscape of Human Blood Cell Trait Variation and Links to Common Complex Disease. *Cell* **167**, 1415-1429 (2016).

2 Chen, L. *et al.* Genetic Drivers of Epigenetic and Transcriptional Variation in Human Immune Cells. *Cell* **167**, 1398-1414 (2016).
