# Supplementary material for: Functional annotation of genetic associations by transcriptome-wide association analysis provides insights into neutrophil development regulation
Source: Commun Biol. 2020 Dec 18;3:790. doi: 10.1038/s42003-020-01527-7 (PMC7749173; doi:10.1038/s42003-020-01527-7)
Supplement: Supplementary file 2 — Description of Additional Supplementary Files [file 42003_2020_1527_MOESM2_ESM.pdf]

Description of Additional Supplementary Files

File Name: Supplementary Data 1

Description:

- Table S1. TWAS hit genes of NEUT# (neutrophil count)
- Table S2. spTWAS associations for NEUT#
- Table S3. TWAS hit genes of NEUT# (neutrophil count)
- Table S4. SgRNA spacer sequence
- Table S5. Primer sequnce used for PCR
