# Supplementary material for: Functional annotation of genetic associations by transcriptome-wide association analysis provides insights into neutrophil development regulation
Source: Commun Biol. 2020 Dec 18;3:790. doi: 10.1038/s42003-020-01527-7 (PMC7749173; doi:10.1038/s42003-020-01527-7)
Supplement: Supplementary file 4 — Reporting Summary [file 42003_2020_1527_MOESM4_ESM.pdf]

nature research

Corresponding author(s): Dr. Shuquan Rao, Dr. Lu Chen

Last updated by author(s): Nov 8, 2020

Reporting Summary

Nature Research wishes to improve the reproducibility of the work that we publish. This form provides structure for consistency and transparency in reporting. For further information on Nature Research policies, see our Editorial Policies and the Editorial Policy Checklist.

Statistics

For all statistical analyses, confirm that the following items are present in the figure legend, table legend, main text, or Methods section.

n/a	Confirmed	
☐	☒	The exact sample size (*n*) for each experimental group/condition, given as a discrete number and unit of measurement
☐	☒	A statement on whether measurements were taken from distinct samples or whether the same sample was measured repeatedly
☐	☒	The statistical test(s) used AND whether they are one- or two-sided<br>*Only common tests should be described solely by name; describe more complex techniques in the Methods section.*
☐	☒	A description of all covariates tested
☐	☒	A description of any assumptions or corrections, such as tests of normality and adjustment for multiple comparisons
☐	☒	A full description of the statistical parameters including central tendency (e.g. means) or other basic estimates (e.g. regression coefficient) AND variation (e.g. standard deviation) or associated estimates of uncertainty (e.g. confidence intervals)
☐	☒	For null hypothesis testing, the test statistic (e.g. *F*, *t*, *r*) with confidence intervals, effect sizes, degrees of freedom and *P* value noted<br>*Give P values as exact values whenever suitable.*
☒	☐	For Bayesian analysis, information on the choice of priors and Markov chain Monte Carlo settings
☐	☒	For hierarchical and complex designs, identification of the appropriate level for tests and full reporting of outcomes
☐	☒	Estimates of effect sizes (e.g. Cohen's *d*, Pearson's *r*), indicating how they were calculated

Our web collection on statistics for biologists contains articles on many of the points above.

Software and code

Policy information about availability of computer code

Data collection	BD FACSDivaTM software was used to acquire data
Data analysis	TWAS and splice TWAS analysis: FUSION (http://gusevlab.org/projects/fusion/)<br>Compute feature weights: GCTA (http://cnsgenomics.com/software/gcta/) and GEMMA (http://www.xzlab.org/software.html)<br>Genotyping data filtering: PLINK (https://www.cog-genomics.org/plink2/)<br>Genetic correlation analysis: Ldsc v1.0.1 (https://github.com/bulik/ldsc)<br>eQTL and sQTL analysis: MatrixEQTL (https://cran.r-project.org/web/packages/MatrixEQTL/index.html)<br>TF enrichment analysis: Lisa (http://lisa.cistrome.org/)<br>GO enrichment and KEGG pathway analysis: DAVID (https://david.ncifcrf.gov/summary.jsp)<br>RNA-seq data analysis pipeline: Trimmomatic software (https://github.com/timflutre/trimmomatic) ; STAR software (https://github.com/alexdobin/STAR; GenomicAlignments R packge (https://bioconductor.org/packages/release/bioc/html/GenomicAlignments.html); DESeq2 R pacakage (http://bioconductor.org/packages/release/bioc/html/DESeq2.html)<br>Combat batch effect correction: sva R package (https://bioconductor.org/packages/release/bioc/html/sva.html)<br>Hidden effects adjustment: PEER (https://github.com/PMBio/peer/wiki)<br>Other statistical analysis: R 3.6.0 (https://www.r-project.org/)<br>Plots and figures: R 3.6.0 ; ggplot2 (https://cran.r-project.org/web/packages/ggplot2/index.html); GraphPad Prism 8 ; adobe illustrator.

For manuscripts utilizing custom algorithms or software that are central to the research but not yet described in published literature, software must be made available to editors and reviewers. We strongly encourage code deposition in a community repository (e.g. GitHub). See the Nature Research guidelines for submitting code & software for further information.

Data

Policy information about availability of data

All manuscripts must include a data availability statement. This statement should provide the following information, where applicable:

- Accession codes, unique identifiers, or web links for publicly available datasets
- A list of figures that have associated raw data
- A description of any restrictions on data availability

Neutrophil count GWAS summary data: ftp://ftp.sanger.ac.uk/pub/project/humgen/summary_statistics/human/2017-12-12/hematological_traits/neut/
Blueprint Whole- genome sequencing data accession number: EGAD00001002663.
CD16+ neutrophil RNA-seq data accession number: EGAD00001002675.
14 human hematopoietic cell RNA-seq data accession number: hematopoietic stem cell (HSC, accession number EGAD00001002316), MPP (multipotent progenitor cell, accession number EGAD00001002363), common lymphoid progenitor (CLP, accession number EGAD00001002489), common myeloid progenitor (CMP, accession number EGAD00001002478), GMP (granulocyte-monocyte progenitor, accession number EGAD00001002306), megakaryocyte-erythroid progenitor cell (MEP, accession number EGAD00001002433), erythroblast (EB, accession number EGAD00001002358), megakaryocyte (MK, accession number EGAD00001002339), neutrophil (accession number EGAD00001002409), monocyte (accession number EGAD00001002308), B lymphocyte (accession number EGAD00001002438), CD4+ T cell (accession number EGAD00001002348), CD8+ T cell (accession number EGAD00001002295), nature killer cell (NK cell, accession number EGAD00001002321).
Normalized expression data of 15 human hematopoietic cell types were downloaded from BloodSpot (http://servers.binf.ku.dk/bloodspot/, HemaExplorer dataset).
All data are available upon request.

Field-specific reporting

Please select the one below that is the best fit for your research. If you are not sure, read the appropriate sections before making your selection.

☒ Life sciences ☐ Behavioural & social sciences ☐ Ecological, evolutionary & environmental sciences

For a reference copy of the document with all sections, see nature.com/documents/nr-reporting-summary-flat.pdf

Life sciences study design

All studies must disclose on these points even when the disclosure is negative.

Sample size	196 individuals were included for neutrophil RNA-seq data generation, 173,480 individuals were included in the blueprint GWAS research.
Data exclusions	Expression prediction models for pseudogenes and MHC region annotated in Gencode V19 were not included in further TWAS analysis .
Replication	Experiments were successfully replicated. The findings reported here were consistent across multiple biological and technical replicates with N reported in each figure legend.
Randomization	For experiments using human CD34+ HSPCs, randomization was not possible; however, proper controls were used and listed for each experimental manipulation.
Blinding	Blinding is not relevant to this study.

Reporting for specific materials, systems and methods

We require information from authors about some types of materials, experimental systems and methods used in many studies. Here, indicate whether each material, system or method listed is relevant to your study. If you are not sure if a list item applies to your research, read the appropriate section before selecting a response.

Materials & experimental systems

n/a	Involved in the study	
☐	☒	Antibodies
☐	☒	Eukaryotic cell lines
☒	☐	Palaeontology and archaeology
☒	☐	Animals and other organisms
☒	☐	Human research participants
☒	☐	Clinical data
☒	☐	Dual use research of concern

Methods

n/a	Involved in the study	
☒	☐	ChIP-seq
☐	☒	Flow cytometry
☒	☐	MRI-based neuroimaging

Antibodies

Antibodies used	anti-human CD34 (581), anti-human CD33 (P67.6), and anti-human CD16 (3G8).
Validation	All antibodies used are commerically available and validated by the vendor for the assay (immunofluorescence) and species used in this study.

Eukaryotic cell lines

Policy information about cell lines

Cell line source(s)	Human CD34+ HSPCs from mobilized peripheral blood of deidentified healthy donors were purchased from AllCells. CD34+ HSPCs were cultured in StemSpan SFEM medium (StemCell Technologies, Vancouver, Canada) supplemented with 1x StemSpan CD34+ expansion supplement (Cat# 02691, STEMCELL Technology). To induce neutrophil differentiation, the cytokine cocktail of 3 ng/mL G-CSF (unless concentration otherwise indicated), 5 ng/mL IL-3, 100 ng/mL FLT3-Ligand, and 50 ng/ml SCF was supplemented to the culture media for 10 days (+/-1 day) before analysis. GM-CSF of 5 ng/ml was added to the culture media additionally in the first 4 days. All these cytokines were of human origin and purchased from PeproTech (Rocky Hill, NJ).
Authentication	N/A
Mycoplasma contamination	N/A
Commonly misidentified lines (See ICLAC register)	N/A

Flow Cytometry

Plots

Confirm that:

☒ The axis labels state the marker and fluorochrome used (e.g. CD4-FITC).

☒ The axis scales are clearly visible. Include numbers along axes only for bottom left plot of group (a 'group' is an analysis of identical markers).

☒ All plots are contour plots with outliers or pseudocolor plots.

☒ A numerical value for number of cells or percentage (with statistics) is provided.

Methodology

Sample preparation	Cells were stained with combinations of antibodies in FACS buffer (PBS + 1% BSA), and then washed twice with FACS buffer.
Instrument	LSRII or LSR Fortessa (BD Biosciences)
Software	BD FACSDivaTM Software and FLowJo (version 10)
Cell population abundance	Cell population percentage is between ~0.5% - 80%
Gating strategy	Gating strategy can be found in the main text and supplementary materials. Basically, FSC/SSC for single live cells, and then marker gene specific cell populations.

☐ Tick this box to confirm that a figure exemplifying the gating strategy is provided in the Supplementary Information.
